# The PVAT–MAMs Axis in Atherosclerosis: A Hypothesis-Driven Cross-Scale Conceptual Framework

**DOI:** 10.3390/ijms27072998

**Published:** 2026-03-25

**Authors:** Sixiang Zhang, Yuhan Li, Yingrui Wang, Bingqi Guo, Zixuan Liu, Zheng Liu

**Affiliations:** The First Clinical Medical College, Shandong University of Traditional Chinese Medicine, Jinan 250013, China; 13831024217@163.com (S.Z.);

**Keywords:** atherosclerosis, perivascular adipose tissue, mitochondria-associated endoplasmic reticulum membranes, mitochondrial signaling, metabolic homeostasis, inflammation, metabolic disease

## Abstract

Atherosclerosis (AS) is the leading cause of cardiovascular disease worldwide, yet its clinical heterogeneity and close association with metabolic disorders are not fully explained by the classical “endothelial injury–lipid deposition–inflammatory amplification” paradigm. In this review, we introduce the PVAT–MAMs axis as a hypothesis-driven, cross-scale conceptual framework linking extravascular metabolic dysfunction to intracellular stress signaling in vascular cells. We propose that, under metabolic stress, dysfunctional perivascular adipose tissue (PVAT) may influence mitochondria-associated endoplasmic reticulum membranes (MAMs) via the release of inflammatory, lipotoxic, and oxidative mediators. Accumulating experimental and associative evidence suggests that perturbation of MAMs is associated with dysregulated calcium handling, lipid metabolism, inflammatory signaling, and redox imbalance, processes implicated in AS progression. Although direct causal relationships remain to be fully established. By synthesizing current findings, this framework provides an integrative perspective on disease heterogeneity and highlights testable pathogenic nodes spanning from PVAT to subcellular MAMs. Finally, we discuss how this conceptual axis may inform hypothesis-driven therapeutic strategies. Importantly, the PVAT–MAMs axis is presented as a hypothesis-driven conceptual model rather than an established signaling pathway, and its mechanistic architecture requires rigorous experimental and translational validation.

## 1. Introduction

Atherosclerosis (AS), the leading cause of cardiovascular disease worldwide, is traditionally explained by the “endothelial injury–lipid deposition–inflammatory amplification” paradigm [1,2]. However, this framework does not adequately explain the marked heterogeneity of plaque phenotypes among individuals with similar lipid burdens, nor the pronounced acceleration of AS associated with metabolic disorders such as obesity and type 2 diabetes (T2D) [3]. These observations indicate that AS pathogenesis extends beyond luminal events and involves regulatory interactions across multiple biological scales.

Two lines of evidence are particularly relevant in this context. First, perivascular adipose tissue (PVAT) has been redefined from a passive structural filler to a dynamic paracrine and endocrine organ [4]. Under metabolic stress, PVAT undergoes a pathogenic phenotypic switch, becoming a source of free fatty acids (FFAs), reactive oxygen species (ROS), and pro-inflammatory cytokines, all of which have been implicated in vascular dysfunction [5,6]. In parallel, on the subcellular level, mitochondria-associated endoplasmic reticulum membranes (MAMs) have emerged as crucial signaling hubs that integrate calcium flux, lipid metabolism, and inflammatory responses in vascular cells, with growing evidence linking MAMs dysregulation to AS-related cellular injury [7,8].

Despite these advances, PVAT dysfunction and MAMs alterations have largely been investigated as independent processes [9,10,11]. Most existing studies adopt a reductionist approach, focusing either on tissue-level paracrine signaling from PVAT or on intracellular stress integration within vascular cells. As a result, potential cross-scale interactions linking the perivascular metabolic environment to organelle-level stress responses remain insufficiently explored.

In this context, the present review adopts an integrative and hypothesis-driven approach that conceptualizes a PVAT–MAMs axis as a potential mechanistic bridge between tissue-level metabolic stress and subcellular signaling integration. Rather than describing isolated pathways, this framework synthesizes experimental and associative evidence across biological levels to explore how PVAT-derived metabolic and inflammatory signals may influence MAMs-mediated regulation of calcium handling, lipid metabolism, oxidative stress, and inflammatory signaling in vascular cells.

By organizing existing evidence within this conceptual framework, the PVAT–MAMs axis offers a systems-level perspective for understanding disease heterogeneity and metabolic acceleration in AS. At the same time, this model remains exploratory and highlights key mechanistic gaps that require further experimental validation and translational investigation.

## 2. The Phenotypic Switch of PVAT: Establishing the Upstream Signal Source in the PVAT–MAMs Axis

### 2.1. PVAT Phenotypes: From Vascular Protection to Atherogenic Driver

PVAT, recognized as the functional “fourth layer” of the vessel wall attached to the adventitia [12], is composed of adipocytes, immune cells, fibroblasts, and progenitors [13]. The adipocytes are phenotypically heterogeneous, encompassing white (WAT), brown (BAT), and beige (BeAT) subtypes, with the balance among them dictating PVAT’s overall function [14,15]. As the proposed upstream initiator of the PVAT–MAMs axis, its anatomical proximity and phenotypic plasticity enable it to directly influence the vascular wall. Under physiological conditions, PVAT mainly exhibits a “beiging” phenotype. Adipocytes with this phenotype are rich in mitochondria and highly express uncoupling protein 1 (UCP1) [16,17]. This process is mainly co-regulated by peroxisome proliferator-activated receptor γ (PPARγ) and its key coactivator, PPARγ coactivator-1α (PGC-1α), which promotes mitochondrial biogenesis and drives the decomposition of FFAs to produce heat [6,18].

Functionally, PVAT releases protective factors through paracrine pathways, including adipose-derived relaxing factors (ADRFs), APN, nitric oxide (NO), fibroblast growth factor 21 (FGF21), Neuregulin 4 (NRG4),which are involved in vasodilation, anti-inflammatory responses, and metabolic regulation, thereby maintaining vascular homeostasis [9,19,20,21]. Furthermore, PVAT acts as a metabolic buffer by uptake and oxidizing circulating FFAs, preventing perivascular lipotoxicity [22,23].

In contrast, metabolic disorders such as obesity and T2D trigger phenotypic “whitening,” characterized by adipocyte hypertrophy, mitochondrial dysfunction, reduced UCP1, diminished APN, and increased release of FFAs, ROS, and pro-inflammatory cytokines (e.g.,Interleukin-6,IL-6, Tumor Necrosis Factor-α,TNF-α) [24,25,26,27]. In T2D, the persistent hyperglycemic environment will further exacerbate this dysfunction by inhibiting key regulatory factors such as PPARγ28. This transition converts PVAT from a protective shield to a potent source of atherogenic signals, with core differences summarized in (Table 1).

This “whitening” phenotypic transformation converts PVAT from a protective tissue into a pathogenic secretory organ that continuously releases a cocktail of deleterious signals. Under pro-inflammatory conditions, PVAT-derived cytokines (e.g., IL-6, TNF-α) and AGEs promote monocyte adhesion and macrophage infiltration [33,38]. A self-sustaining vicious cycle is established when plaque-derived IL-1β feeds back to further amplify PVAT inflammation [39]. This bidirectional inflammatory crosstalk is enabled by the close anatomical adjacency between PVAT and the vascular wall, which forms the structural basis for PVAT’s regulation in AS. Histological studies in humans have confirmed significant infiltration of macrophages and lymphocytes in the PVAT surrounding coronary atherosclerotic plaques, providing direct evidence for this self-reinforcing inflammatory loop [40,41]. Concurrently, in a state of lipotoxic signaling, the excessive release of FFAs induces endothelial dysfunction, partly through uncoupling endothelial nitric oxide synthase (eNOS), and activates pro-inflammatory pathways in vascular cells [42,43]. ROS derived from activated NADPH oxidase (NOX) and dysfunctional mitochondria within PVAT constitute a major source of perivascular oxidative stress [44]; these reactive species promote LDL oxidation, exacerbate inflammation, and inflict direct vascular damage [45,46]. Furthermore, PVAT drives vascular remodeling by releasing factors (e.g., PDGF) that, together with inflammatory cues, stimulate VSMCs to switch from a contractile to a synthetic phenotype [34,35]. This transition is characterized by upregulated expression of matrix metalloproteinases (e.g., MMP-2/9), thereby promoting plaque instability [47]. Crucially, these PVAT-derived signals converge on intracellular organelles, particularly MAMs, to disrupt core cellular homeostatic functions.

### 2.2. Molecular Driving Mechanisms of PVAT Phenotypic Transition: Distinct Roles of Obesity, T2D, and AS

The pathogenic transformation of PVAT from a protective organ into a persistent source of atherogenic signals is not spontaneous but is actively driven by the converging pathophysiological milieus of obesity, T2D, and AS itself. Each condition imposes distinct stresses that collectively reprogram PVAT. This reprogramming operates across multiple tiers: it involves transcriptional rewiring within PVAT, deterioration of the local metabolic microenvironment, and integration of systemic mechanical and neuroendocrine imbalances [48,49,50]. Together, these disturbances lock PVAT into a stable, dysfunctional state.

In obesity, the chronic energy surplus alters PVAT through mass effect and immune remodeling. Adipocyte hypertrophy and hyperplasia lead to tissue expansion and local hypoxia [51]. This hypoxic environment stabilizes and induces the abnormal accumulation of HIF-1α. The accumulated HIF-1α further activates signaling pathways such as Notch, directly inhibiting the expression of thermogenic genes like UCP1 and promoting lipid storage rather than oxidation [52]. Concurrently, the immune landscape within PVAT undergoes a profound pro-inflammatory shift. Macrophages polarize towards the M1 state, secreting cytokines that exacerbate insulin resistance and vascular dysfunction [53,54]. Increased neutrophil infiltration drives macrophage activation by releasing CCL2 and TNF-α [55], along with increased infiltration of dendritic cells and mast cells [56,57]. This further amplifies this inflammatory environment, while the number of eosinophils, which have a metabolically stabilizing effect, decreases [58]. Thus, obesity transforms PVAT into a chronically inflamed, dysfunctional adipose depot.

In T2D, high blood sugar causes oxidative stress, which activates the pro-inflammatory Nuclear Factor Kappa B (NF-κB) pathway in PVAT [59,60]. More specifically, the engagement of AGEs with RAGE triggers a surge of mitochondrial ROS. These ROS inhibit the deacetylase silent information regulator 2 homolog 1 (SIRT1), a crucial regulator of mitochondrial health. Impaired SIRT1 function leads to the deactivation of PGC-1α, the key transcriptional coactivator required for maintaining the beiging program and mitochondrial biogenesis [61]. This double blow of inflammation and impaired antioxidant defense can damage the protective metabolic phenotype of PVAT.

As AS progresses, the diseased vascular wall will actively feedback, exacerbating the dysfunction of perivascular fat. On one hand, the lipid-rich plaque microenvironment releases oxidized low-density lipoprotein (ox-LDL), which binds to the LOX-1 receptor on PVAT cells. The activated intracellular pathways, in collaboration with NF-κB, inhibit the protective PPARγ/PGC-1α transcriptional complex, locking PVAT in a whitening state [62]. Furthermore, the abnormal biomechanical environment of narrowed arteries (such as changes in shear stress) specifically activates the mechanically sensitive Piezo1 channels in PVAT [63]. The resulting calcium influx stimulates the YAP/TAZ signaling pathway, directly driving the transformation of PVAT towards a pro-fibrotic and pro-inflammatory phenotype, thereby creating a perivascular microenvironment that further accelerates plaque growth [64,65].

While initiated by distinct triggers, the pathogenic pathways of obesity, T2D, and AS converge on a common outcome: the stable silencing of PVAT’s protective, beiging program and its lock-in into a persistent, signal-releasing pathological state. This process is further reinforced by systemic factors. For instance, the sympathetic nervous system tone, which normally stimulates beneficial PVAT thermogenesis via β3-adrenergic receptors, is often reduced in these metabolic disorders, removing a crucial brake on dysfunction [66,67]. Aging adds another layer of vulnerability through epigenetic modifications that further suppress PGC-1α expression [68]. Together, these drivers ensure that PVAT becomes a resilient and active contributor to the AS cascade, setting the stage for its long-range communication with vascular cell organelles.

## 3. MAMs: The Intracellular Effector and Integrator in the PVAT–MAMs Axis

If dysfunctional PVAT is the source of atherogenic signals, then MAMs function as the critical subcellular effector and integration hub within the PVAT–MAMs axis. The homeostatic integrity of this hub is paramount for preserving vascular homeostasis and counteracting the progression of AS.

### 3.1. Core Structural and Functional Overview of MAMs

Mitochondria-associated endoplasmic reticulum membranes are dynamic, 10–30 nm membrane contact sites that function as a central signaling hub in vascular cells, integrating calcium homeostasis, lipid metabolism, and mitochondrial quality control [7,69]. Their structural integrity is maintained by core tethering complexes, including Mitofusin 2 (MFN2), the Vesicle-Associated Membrane Protein-Associated Protein B (VAPB)–Protein Tyrosine Phosphatase Interacting Protein 51 (PTPIP51) pair, and the Inositol 1,4,5-trisphosphate receptor (IP_3_R)–Glucose-regulated protein 75 (GRP75)–Voltage-dependent anion channel 1(VDAC1) complex. These complexes create specialized microdomains that are packed with functional proteins. These include Ca^2+^ channels, lipid-metabolizing enzymes (e.g., Acyl-CoA Synthetase 4, ACSL4), and stress sensors (e.g., Protein Kinase R-like Endoplasmic Reticulum Kinase, PERK and the Sigma-1 Receptor, Sig-1R) [7]. The activity of MAMs is not static; it is fine-tuned through post-translational modifications that allow it to respond to the cell’s metabolic needs [7]. However, under sustained metabolic stress, this precise regulation fails. The ensuing MAMs dysfunction, characterized by calcium overload, disrupted lipid handling, and the initiation of oxidative stress and inflammation, becomes a key pathological event driving disease progression.

### 3.2. MAMs as a Protective Switch for Vascular Homeostasis

Under physiological conditions, MAMs function as a crucial “protective switch” for regulatory vascular homeostasis by integrating and fine-tuning cellular stress responses [70]. This role is achieved through the precise coordination of several core functions at the ER-mitochondria interface.

#### 3.2.1. Orchestrating Ca^2+^ for Energy and Safety

The IP_3_R–GRP75–VDAC1 complex at MAMs forms a channel that directs Ca^2+^ from the ER to mitochondria [71,72]. This serves two vital purposes: it fuels ATP production to support functions like vascular contraction, and it prevents harmful Ca^2+^ buildup in the cytoplasm, thereby safeguarding against protease activation and eNOS uncoupling, and the triggering of aberrant cell death pathways (apoptosis), thus maintaining vascular relaxation [71,72]. The Sig-1R at MAMs stabilizes this Ca^2+^ channel under stress, preventing leaks and ensuring efficient transport [73].

#### 3.2.2. Managing Lipids to Prevent Toxicity

MAMs regulate lipid flow to prevent toxic damage. Proteins like Oxysterol-binding Protein-related Protein 5 and 8 (ORP5/8) exchange phosphatidylserine (PS) and phosphatidylcholine (PC) to preserve membrane integrity [74,75]. The enzyme Acyl-CoA: cholesterol acyltransferase 1(ACAT1) esterifies free cholesterol (FC) to cholesteryl ester (CE) in response to rising local levels, preventing excessive CE accumulation and foam cell formation while maintaining a CE reserve [76,77]. The MAMs protein GRAM Domain Containing 1C(GRAMD1C) balances cholesterol between the ER and mitochondria, preventing its buildup inside mitochondria where it would block energy production and trigger ROS overproduction, a major source of oxidative stress [78,79].

#### 3.2.3. Responding to Stress to Promote Survival

During mild ER stress (ERS), the MAMs-localized protein PERK is activated. It temporarily reduces global protein synthesis while boosting repair protein expression, rebalancing cellular protein homeostasis [80,81]. A key aspect of this response is the stabilization of ER-mitochondria contacts by PERK, which serves to restrict pathological calcium transfer to mitochondria, thus preventing calcium overload, mitochondrial permeability transition pore (mPTP) opening, and the initiation of apoptosis [82,83]. This calcium-buffering action is synergistically reinforced by Bcl-2 family proteins resident at MAMs, which directly interact with and restrain the IP_3_R and VDAC1 channels, further limiting pro-death calcium signaling and blocking a critical step in the apoptotic cascade [84,85]. By coordinately ensuring calcium homeostasis and mitochondrial integrity, this MAMs-centric stress response constitutes a critical cellular barrier against metabolic dysfunction and premature cell death, thereby also mitigating a key trigger for inflammatory activation.

#### 3.2.4. Controlling Quality: Repair or Remove

MAMs coordinate a tiered mitochondrial quality control system that is precisely calibrated to the degree of damage. In cases of mild impairment, repair is facilitated through mitochondrial fusion, mediated by the MAMs protein MFN2. This process dilutes damaged components and is vital for VSMC function [86]. MFN2 additionally curbs excessive fission by impeding Dynamin-related protein 1 (Drp1) recruitment86. For severe damage, MAMs initiate a selective autophagic clearance program known as mitophagy, such as via the PINK1- Parkin RBR E3 Ubiquitin Protein Ligase (Parkin) pathway [87,88]. The MAMs receptor FUNDC1 serves as a critical decision-maker in this process [89,90]. Normally inactivated by phosphorylation, FUNDC1 becomes dephosphorylated under stress. Crucially, dephosphorylated FUNDC1 preferentially promotes fusion-mediated repair over mitophagy when damage is limited, thereby preventing the premature clearance of functional organelles [89,90]. This precise stratification of response, in which fusion addresses mild damage and autophagy addresses severe damage, ensures the mitochondrial network remains robust to meet vascular energy demands.

#### 3.2.5. Adaptive Versus Maladaptive Roles of MAM Dynamics

It is essential to emphasize that MAMs are not intrinsically pathogenic structures. Rather, they function as dynamic signaling hubs that integrate endoplasmic reticulum and mitochondrial activities to coordinate cellular responses to metabolic demands and stress [7]. Under physiological conditions, MAMs play indispensable roles in calcium homeostasis, lipid exchange, mitochondrial bioenergetics, and quality control [69]. Consequently, moderate or transient remodeling of ER–mitochondria contacts may represent an adaptive cellular response aimed at restoring homeostasis under mild or acute stress.

In the context of vascular biology, such adaptive remodeling may initially support increased mitochondrial energy production in ECs or VSMCs exposed to fluctuating metabolic conditions, or facilitate protective autophagy to clear damaged organelles. However, when metabolic disturbances become chronic, which occurs in obesity, type 2 diabetes, or persistent perivascular inflammation, this adaptive response can be overwhelmed, and MAM interactions may become excessive, sustained, and dysregulated. Prolonged ER–mitochondria coupling can promote pathological mitochondrial calcium overload, sustained oxidative stress, aberrant lipid accumulation, and chronic inflammasome activation, ultimately driving cellular dysfunction and contributing to atherosclerosis progression [91].

This adaptive–maladaptive continuum, in which early, transient MAM remodeling reflects compensatory attempts to maintain cellular homeostasis under escalating metabolic pressure, while sustained or exaggerated remodeling tips the balance toward maladaptive signaling, is consistent with the broader concept of cellular hormesis: low-level stress triggers protective responses that become harmful when stress becomes chronic or severe. Recognizing this duality is critical for interpreting the potential role of the PVAT–MAMs axis in atherosclerosis, as it implies that MAMs-directed interventions must be carefully timed and calibrated [92]. Defining the temporal trajectory and molecular thresholds that mark the transition from adaptive to maladaptive MAMs remodeling is therefore a key priority for future research and for identifying appropriate therapeutic windows.

## 4. Core Mechanisms of the PVAT–MAMs Axis: Integrating Upstream Signals and Downstream Dysfunction

### 4.1. The PVAT–MAMs Axis: From Tissue-Level Dysfunction to Organelle-Level Pathogenesis

Classical theory attributes the initiation of AS to endothelial dysfunction, lipid infiltration, and inflammation triggered by risk factors [1]. The PVAT plays an active role in this process. Under metabolic disorder, its released inflammatory factors and chemokines serve as key paracrine signals that initiate endothelial activation, recruit monocytes/macrophages, and drive early plaque formation [20,93,94]. However, the pathogenesis of AS extends beyond intercellular communication. A pivotal question remains: how do systemic metabolic disturbances translate into sustained cellular damage within the vascular wall?

This section presents a conceptual synthesis rather than a validated signaling pathway. The proposed PVAT–MAMs axis is intended to align existing, largely indirect evidence across biological scales, rather than to assert a definitive or exclusive mechanistic route.

Recent research points to a deeper, “cross-scale” mechanism whereby the external vascular microenvironment directly remodels intracellular organelle networks. A landmark study by Zhang et al. provides strong experimental evidence that systemic metabolic stress is translated into organelle-level restructuring [95]. They demonstrated that a high-fat, high-cholesterol diet, or direct cholesterol treatment in vitro, specifically enhances endoplasmic reticulum-mitochondria contact and increases the number of MAMs in VSMCs [95]. Functionally, these augmented MAMs act as hyperactive calcium conduits, leading to pathological mitochondrial calcium overload. This mitochondrial dysfunction is further linked to intracellular lipid deposition, a process that may involve the downregulation of FFA β-oxidation (e.g., via acyl-CoA dehydrogenase medium chain, ACADM). Crucially, the study demonstrated a causal relationship through genetic manipulation: artificially promoting MAMs formation exacerbated the atherogenic phenotype, whereas inhibiting a key tethering protein (PACS2) alleviated it [95]. This work supports the view that MAMs augmentation represents a critical mechanistic link between dietary stress and atherosclerotic progression.

Lipid stress (e.g., cholesterol) has been shown to induce MAMs structural and functional dysregulation, which is associated with calcium overload and metabolic dysfunction implicated in AS. To complete this cross-scale narrative, the physiologically relevant upstream signal source must be identified. Within the anatomical context of AS, dysfunctional PVAT represents a plausible and persistent local source of such lipotoxic signals among several potential contributors. The “whitening” transformation of PVAT converts it from a metabolic buffer into a chronic emitter of FFAs, cholesterol derivatives, and inflammatory factors, thereby creating a microenvironment comparable to conditions reported to induce pathogenic MAMs remodeling [95]. This inference is further supported by cell biological evidence that saturated FFAs (e.g., palmitate) can directly impair MAMs integrity [96]. Therefore, we synthesize this evidence to construct the “PVAT–MAMs axis” framework. Within this framework, sustained PVAT dysfunction may represent one of several tissue-level sources of metabolic stress capable of influencing intracellular vulnerability in vascular cells, thereby potentially contributing to AS progression.

It should be emphasized that MAMs are not intrinsically pathogenic structures. Under physiological conditions, ER–mitochondria contacts support calcium signaling, lipid metabolism, mitochondrial bioenergetics, and adaptive stress responses. Accumulating evidence indicates that the functional consequences of MAMs remodeling are context- and cell-type-dependent: while moderate or transient enhancement may facilitate metabolic adaptation, sustained or dysregulated expansion under chronic metabolic stress is more likely to bias vascular cells toward inflammatory and lipotoxic states [7]. Accordingly, the pathogenic role of MAMs discussed here should be interpreted as conditional rather than universal.

Importantly, although this review highlights PVAT as a salient local source of metabolic stress signals, AS is driven by multiple parallel mechanisms, including luminal lipid infiltration, hemodynamic forces, and systemic immune activation [1]. The proposed PVAT–MAMs axis therefore does not replace these classical paradigms but provides a complementary perivascular framework through which adipose-derived stress may be sensed and integrated at subcellular organelle interfaces.

Conceptually, the PVAT–MAMs axis should be regarded as a modular and context-dependent signaling network rather than a fixed unidirectional pathway. Distinct PVAT depots, such as thermogenic-dominant thoracic PVAT, lipotoxic-expanded abdominal PVAT, and inflammation-prone coronary PVAT, likely engage this axis through partially divergent mechanisms, potentially contributing to the regional distribution of AS [4,97,98].

### 4.2. Anatomical and Biophysical Considerations in PVAT-to-Vessel Communication

This cross-scale signaling is anatomically grounded in the close adhesion of PVAT to the vascular adventitia. The adventitia, media, and intima form layered diffusion barriers that limit the unrestricted penetration of soluble mediators. Therefore, the proposed PVAT–MAMs linkage likely mediates stage- and context-dependent stress transmission.

#### 4.2.1. Local Paracrine and Diffusion-Limited Effects

In early or moderate disease, PVAT-derived mediators likely exert primarily local effects on adjacent adventitial and medial cells. Small lipophilic molecules such as FFAs and certain ROS can influence nearby vascular cells under conditions of sustained exposure and high local concentration [99,100]. They have been shown that saturated FFAs and oxidative stress can alter ER–mitochondria contacts and modify MAMs-associated proteins [96]. However, these findings derive largely from in vitro or systemic metabolic models, and direct in vivo tracking of PVAT-derived factors to MAMs remains lacking. Accordingly, early-stage PVAT signaling is better conceptualized as creating a permissive metabolic and inflammatory microenvironment that may increase intracellular vulnerability, rather than as a directed subcellular transport mechanism.

#### 4.2.2. Receptor-Mediated Stress Amplification

Hydrophilic mediators such as cytokines and AGEs initiate signaling through cell-surface receptors (e.g., TNF receptors, IL-6 receptor complexes, RAGE), activating transcriptional and stress-response pathways including NF-κB and ER stress cascades. Sustained activation of these pathways has been associated with remodeling of ER–mitochondria contacts [101,102,103,104,105]. Sustained activation of these pathways has been associated with remodeling of ER–mitochondria contacts. Rather than implying selective trafficking of signaling complexes to MAMs, it is more plausible that chronic receptor-mediated stress indirectly reshapes intracellular organelle architecture, with MAMs functioning as one stress-sensitive integration site among several intracellular hubs.

#### 4.2.3. Vasa Vasorum as a Structural Amplifier in Advanced Disease

With progression of AS, the architectural landscape of the arterial wall undergoes substantial remodeling. One of the most significant structural changes is the expansion and neovascularization of the vasa vasorum, particularly in advanced and metabolically stressed lesions [106]. This microvascular network extends from the adventitia toward the media and, in severe cases, into the intima, thereby reducing the effective diffusion distance between PVAT and intramural cells [106,107,108]. Under such conditions, transport of soluble mediators is less dependent on passive diffusion across thickened arterial layers and may instead occur through microvascular perfusion pathways. Vasa Vasorum expansion therefore represents a plausible anatomical conduit through which PVAT-derived cytokines, lipids, and oxidative mediators could gain access to deeper vascular compartments. Importantly, Vasa Vasorum neovessels are often immature and structurally fragile [109]. Increased permeability and episodic rupture contribute to intraplaque hemorrhage, erythrocyte infiltration, and local iron deposition, which collectively intensify oxidative and inflammatory stress within the plaque microenvironment [110]. These processes may amplify intramural metabolic stress independently of, or in parallel with, PVAT-derived signals. Thus, rather than serving as direct proof of targeted organelle communication, Vasa Vasorum remodeling may function as a structural amplifier that enhances and sustains perivascular–intramural stress transmission during advanced disease stages.

Collectively, these dissemination routes, particularly the expansion and remodeling of the vasa vasorum in metabolically stressed vessels, may increase the likelihood that PVAT-derived mediators gain sustained access to vascular cells. Once internalized or sensed, such signals may intersect with intracellular stress-integrating platforms, among which MAMs represent one potential hub.

### 4.3. Inflammation Amplification Axis: PVAT-Derived Pro-Inflammatory Signals May Target MAMs to Activate a Self-Sustaining “Inflammation–Injury” Circuit

**Figure 1:**
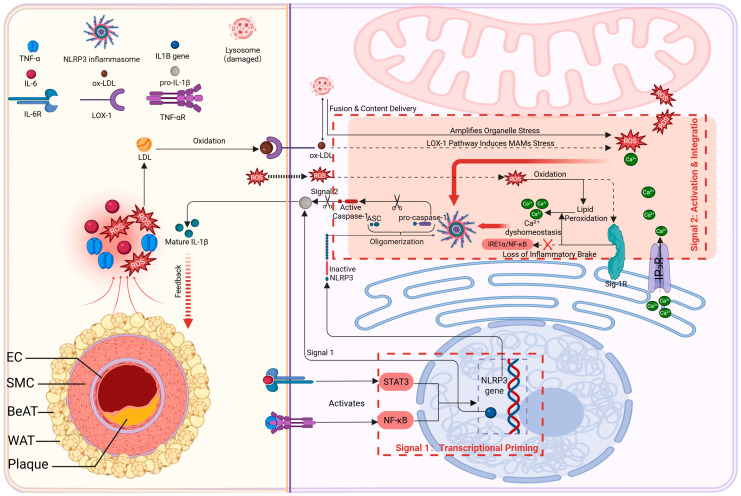
Inflammation axis: Conceptual schematic of the proposed PVAT–MAMs–mediated inflammatory amplification circuit in Atherosclerosis. (**Left**) PVAT as a signal source: Under metabolic stress, PVAT adopts a pro-inflammatory phenotype, releasing cytokines (e.g., TNF-α, IL-6) and ROS. (**Right**) Signal integration at MAMs: These signals converge at MAMs within vascular cells, engaging two concurrent processes: (1) NLRP3 inflammasome activation: Cytokine signaling primes the NLRP3 inflammasome, whose full activation is triggered by MAMs-localized danger signals (e.g., ox-LDL internalized via LOX-1) together with ROS and Ca^2+^ fluxes, associated with IL-1β production. (2) Disruption of homeostatic inhibition: ROS impair the function of the MAMs stabilizer Sig-1R, thereby removing its inhibitory control over the IP_3_R calcium channel and pro-inflammatory pathways (e.g., IRE1α/NF-κB). Apoptosis-Associated Speck-like Protein Containing a CARD (ASC) Created in BioRender. Zhang, S. (2026) https://BioRender.com/o8jrcc6.

Once within vascular cells, these extravascular stress signals may engage intracellular signaling networks that converge at MAMs. Under metabolic stress, PVAT becomes a chronic source of proinflammatory cytokines (IL-6, TNF-α) and ROS that diffuse to the vascular wall [111]. These signals may intersect with MAMs-enriched inflammatory signaling domains, a subcellular platform that has been implicated in NLRP3 inflammasome priming and activation, although alternative or parallel intracellular signaling hubs may also be involved (Figure 1). For example, cytokine–receptor signaling via NF-κB and Signal Transducer and Activator of Transcription 3 Pathway (STAT3) provides the priming signal [112,113,114]. while a second “danger” signal (e.g., OxLDL via LOX-1 together with local MAMs ROS/Ca^2+^) triggers full NOD-Like Receptor Protein 3 (NLRP3) assembly [115,116]. In this way, MAMs may function as an intracellular site where PVAT-derived inflammatory signals are coordinated.

**Figure 2:**
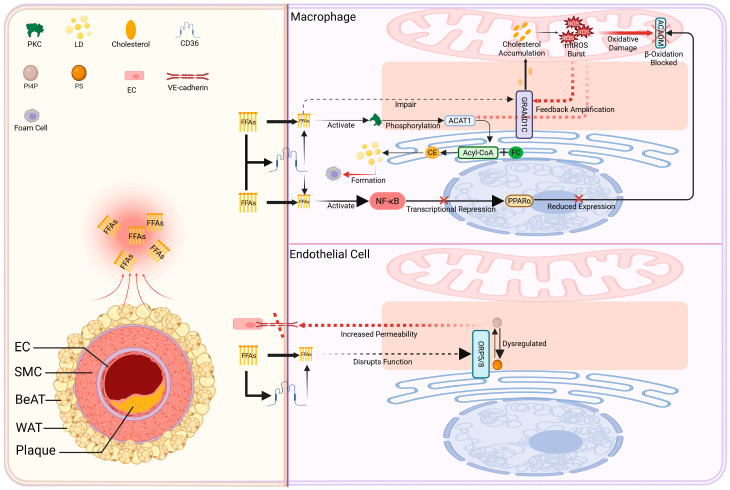
Lipotoxicity axis: Schematic model illustrating the proposed PVAT–MAMs–associated lipotoxic cascade in Atherosclerosis. (**Left**) PVAT as a lipotoxic source: In obesity and T2D, PVAT releases excessive FFAs. (**Right**) MAMs-mediated lipid dysregulation: FFAs target vascular cells (e.g., macrophages, ECs) and converge at MAMs, is proposed to engage three interconnected pathways: (1) Foam cell formation: FFA-activated PKC hyperactivates ACAT1, promoting cholesterol esterification and LD accumulation. (2) Endothelial Barrier Dysfunction: FFAs disrupt the function of the lipid transporters ORP5/8, impairing the exchange of PS and Phosphatidylinositol 4-Phosphate (PI4P) between the ER and mitochondria. This may compromise endothelial membrane integrity and affect the localization and function of VE-cadherin, and increase its permeability. (3) Impaired FFA oxidation: FFAs suppress the MAMs-associated enzyme ACADM via transcriptional and oxidative mechanisms, blocking lipid clearance. (4) Mitochondrial cholesterol dysregulation: FFAs impair GRAMD1C function, leading to intramitochondrial cholesterol accumulation, electron transport chain inhibition, and mtROS overproduction. Created in BioRender. Zhang, S. (2026) https://BioRender.com/ltkjkwb.

**Figure 3:**
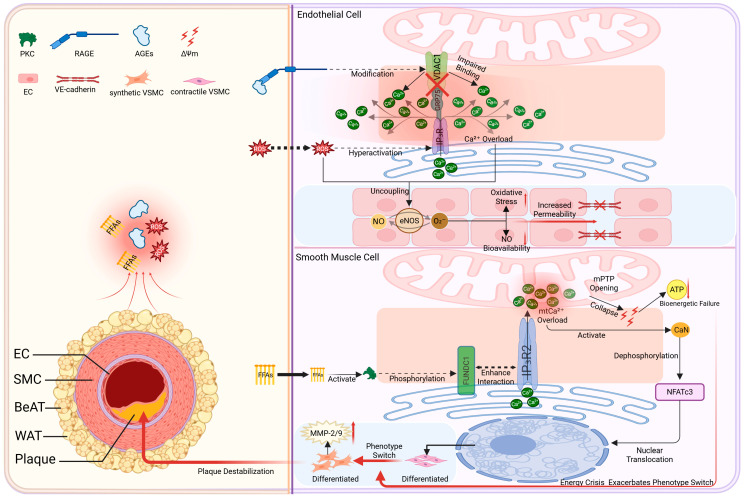
Calcium signaling axis: Schematic overview of proposed mechanisms linking PVAT-derived signals to MAMs-associated calcium signaling dysregulation. (**Left**) Source of signals: In obesity and T2D, PVAT releases ROS, FFAs, and AGEs. (**Right**) MAMs-mediated calcium dysregulation: These signals target vascular cells and converge at MAMs, disrupting calcium handling through two primary mechanisms: (1) Core channel disruption: ROS hyperactivate IP_3_R, while AGEs impair VDAC1-GRP75 binding, disrupting the IP_3_R-GRP75-VDAC1 complex and uncoupling calcium flux. (2) FFA-activated PKC phosphorylates FUNDC1, hypothesized to enhance its interaction with Inositol 1,4,5-Trisphosphate Receptor, Type 2 (IP_3_R2), potentially promoting excessive calcium influx into mitochondria.Downstream consequences: The resultant calcium overload triggers cell-type-specific dysfunction: In ECs, it induces eNOS uncoupling, shifting its enzymatic output from NO to superoxide anion, impairs barrier function. In VSMCs, it activates CaN, facilitating NFATc3 nuclear translocation and a phenotypic switch to a synthetic state, characterized by increased MMP-2/9 expression and plaque destabilization. Mitochondrial calcium overload is associated with mPTP opening and bioenergetic impairment. Created in BioRender. Zhang, S. (2026) https://BioRender.com/flku2v9.

**Figure 4:**
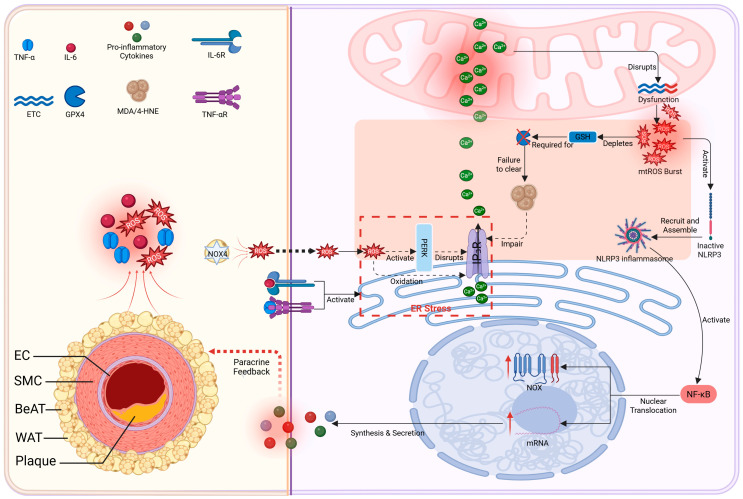
Oxidative stress axis: Conceptual schematic of a proposed self-reinforcing oxidative stress circuit involving the PVAT–MAMs axis in Atherosclerosis. (**Left**) PVAT-derived oxidative insult: In obesity, PVAT generates excessive ROS (e.g., via NOX4) and inflammatory factors. (**Right**) MAMs-centric amplification loop: PVAT-derived signals target vascular cells and converge at MAMs, constituting a proposed multi-step cycle: (1) ER Stress and Calcium Dysregulation: ROS induce ER stress, activating PERK at MAMs and oxidizing the IP_3_R channel, leading to aberrant cytosolic Ca^2+^ release. (2) Mitochondrial ROS Burst: Cytosolic Ca^2+^ is taken up by mitochondria, causing overload and a dramatic burst of mtROS from the impaired electron transport chain. (3) Inflammatory Amplification: mtROS activates NLRP3 inflammasomes and NF-κB, upregulating NOX and cytokines. (4) Antioxidant Failure: The MAMs antioxidant Glutathione Peroxidase 4 (GPX4) is inactivated, and ROS continuously consumes Glutathione (GSH). This impairs the clearance of lipid hydroperoxides, leading to the accumulation of reactive lipid peroxidation products (e.g., Malondialdehyde, MDA, 4-HNE). Created in BioRender. Zhang, S. (2026) https://BioRender.com/s2dq3ul.

The destabilization of MAMs chaperones may further amplifies inflammation. For instance, Sig-1R, normally stabilizes the IP_3_R–GRP75–VDAC1 Ca^2+^ channel, so its impairment disrupts Ca^2+^ homeostasis and unleashes pro-inflammatory signaling (e.g., Inositol-Requiring Enzyme 1α, IRE1α/NF-κB) [117,118,119,120]. PVAT-derived ROS exacerbate this by causing lipid peroxidation at MAMs, which inactivates Sig-1R and other proteins. Inactive Sig-1R thus delivers a double hit: worsened Ca^2+^ overload and loss of the normal brake on inflammatory pathways, thereby favoring a sustained pro-inflammatory state at MAMs. Ultimately, the maturation and secretion of interleukin-1β (IL-1β), mediated by cysteinyl aspartate-specific protease 1 (caspase-1), not only amplifies local vascular inflammation but also is proposed to form a critical positive feedback loop. Secreted IL-1β can act back on PVAT (e.g., via the IL-1 receptor), further entrenching its pro-inflammatory phenotype and thereby potentially sustaining inflammatory signaling within the conceptual PVAT–MAMs framework [121].

(Figure 1, Figure 2, Figure 3 and Figure 4 are intended to present an integrative conceptual framework informed by current evidence. The precise contribution, hierarchy, and temporal dynamics of these interactions in vivo remain to be clarified in future studies. Solid lines indicate mechanisms supported by experimental evidence in vascular or cellular systems. Dashed lines represent proposed or integrative interactions within the conceptual PVAT–MAMs framework that require further validation in vivo).

### 4.4. Lipid Dysregulation Axis: PVAT–FFA–Associated Disruption of MAMs Lipid Metabolism and a Lipotoxic Amplification Cascade

In obesity/T2D, PVAT releases excess FFAs that act as a lipotoxic signal in the PVAT–MAMs axis. These FFAs enter vascular cells via Cluster of Differentiation 36 (CD36) or diffusion [122,123] and accumulate at MAMs, a lipid metabolism hub. There they perturb lipid handling and generate toxic intermediates (Figure 2).

At MAMs, FFAs have been shown to disrupt lipid-handling pathways associated with MAMs: PKC activation leads to ACAT1-mediated cholesterol esterification and lipid droplet/foam cell formation [124,125,126] while FFA disrupts ORP5/8-mediated phospholipid exchange, impairing endothelial barrier function [127,128,129]. Saturated FFAs also block mitochondrial β-oxidation (via ACADM downregulation/oxidation95), worsening lipid overload. These insults provoke a feedback loop: accumulated mitochondrial cholesterol (due to GRAMD1C dysfunction) inhibits the ETC and spikes mitochondrial ROS (mtROS) [130], which in turn further damages MAMs lipid-handling proteins. In sum, lipid deposition at MAMs may become increasingly difficult to resolve, thereby aggravating metabolic dysfunction [116]. This FFA–GRAMD1C–cholesterol–mtROS circuit provides a mechanistic framework that may help explain how lipid deposition, once initiated, can become self-reinforcing and progressively aggravate metabolic dysfunction.

### 4.5. Calcium Signaling Dysregulation Axis: PVAT Signals Disrupt MAMs Calcium Transport, Inducing a “Calcium Overload–Functional Failure” Cascade

Pathological factors released from dysfunctional PVAT may contribute to disturbances in cellular Ca^2+^ homeostasis by targeting key components such as the MAMs-associated calcium transport machinery. These molecules, employing modes ranging from direct diffusion to receptor-mediated actions, ultimately target the core calcium transport machinery at MAMs, specifically the IP_3_R–GRP75–VDAC1 complex (Figure 3) [131,132,133]. PVAT-derived ROS can oxidize the IP_3_R, leading to its hyperactivation [133], while AGEs, via RAGE signaling, modify VDAC1 and impair its binding to GRP75, compromising the tethering complex [134,135]. This dual assault is proposed to uncouple ER–mitochondrial Ca^2+^ flux, leading to cytosolic Ca^2+^ overload and impaired mitochondrial Ca^2+^ uptake.

The consequences of this disrupted Ca^2+^ handling are cell-type-specific yet potentially deleterious. In ECs, Ca^2+^ overload synergizes with oxidative stress to induce eNOS uncoupling, impairing vasodilation and barrier function [136]. In VSMCs, elevated cytosolic Ca^2+^ activates calcineurin (CaN), which dephosphorylates NFATc3, prompting its nuclear translocation. This drives a phenotypic switch to a synthetic state, characterized by upregulation of matrix-degrading enzymes (e.g., MMP-2/9), thereby potentially contributing to plaque destabilization [137,138]. Furthermore, PVAT-derived FFAs exacerbate this dysregulation by activating PKC, which phosphorylates the MAMs protein FUNDC1 [139,140,141]. This phosphorylation is proposed to hijack FUNDC1’s normal function, converting it into a pathological conduit for excessive mitochondrial Ca^2+^ influx [142]. This may promote mitochondrial calcium overload, facilitate mPTP opening, and compromise mitochondrial membrane potential and bioenergetics [143].

### 4.6. Oxidative Stress Axis: A Self-Reinforcing PVAT–MAMs Cycle Potentially Sustains Oxidative Stress in AS

Dysfunctional PVAT generates excess ROS (via NOX4) that may induce ER stress and activate PERK at MAMs, although PERK activation is not exclusive to MAMs [144,145,146,147]. PERK activation and ROS-mediated oxidation of MAMs channels (IP_3_R) cause aberrant Ca^2+^ release, leading to mitochondrial Ca^2+^ overload [146,147]. This may impair electron transport chain function and promote increased superoxide production [148,149], which then activates inflammasomes and NF-κB. The resulting cytokines upregulate vascular NOX and feedback to PVAT, prompting still more ROS [150]. Together, these interactions suggest a vicious cycle in which PVAT-derived ROS may promote mtROS generation, inflammation, and further ROS production (Figure 4).

High ROS levels may compromise MAMs’ antioxidant defenses: glutathione peroxidase 4 (GPX4) becomes inactivated, allowing lipid peroxides (malondialdehyde, MDA and 4-hydroxynonenal, 4-HNE) to accumulate [151,152].These peroxidation products covalently modify MAMs proteins (e.g., IP_3_R, VDAC1), further disrupting Ca^2+^ signaling and enhancing inflammation [153,154].

### 4.7. An Integrated PVAT–MAMs Network Coordinates Atherosclerotic Signaling

Importantly, structural changes such as vasa vasorum expansion may spatially amplify signal delivery, thereby increasing the probability that multiple stress axes converge simultaneously at MAMs. Because calcium channels, lipid enzymes, and inflammasome components are co-localized there [70], may influence or potentiate the others. For example, inflammatory signals enhance local lipid dysregulation, which in turn fuels NLRP3 inflammasome activation. Excess lipids and cholesterol at MAMs may exacerbate inflammatory signaling [92,147]. Similarly, Ca^2+^ and ROS loops feed each other: pathological Ca^2+^ release produces mtROS, and ROS oxidizes the IP_3_R-VDAC1 tether to release more Ca^2+^ [155]. Redox sensors like thioredoxin-interacting protein (TXNIP) at MAMs directly link oxidative stress to NLRP3 activation [156,157]. This mechanism provides a plausible link between oxidative stress and inflammatory signaling within vascular cells. The relative magnitude, temporal dominance, and signal prioritization among these converging pathways remain to be formally quantified in vivo.

### 4.8. Evolving Dynamics of the PVAT–MAMs Axis Across Disease Stages

The interactions between PVAT and vascular MAMs should not be viewed as static mechanisms but rather as dynamic processes that evolve across the natural history of AS. In early, subclinical stages, PVAT may exhibit mild inflammatory activation while retaining partial metabolic flexibility, potentially inducing moderate MAM remodeling that serves adaptive functions such as optimizing mitochondrial bioenergetics and maintaining cellular stress tolerance [92,158]. As disease progresses, however, pronounced PVAT phenotypic switching, which is characterized by macrophage infiltration, sustained cytokine secretion, and enhanced lipolysis, may drive persistent and excessive ER–mitochondria coupling, tipping the balance from adaptation to maladaptive signaling [159,160]. This spatiotemporal framework, which requires rigorous validation through stage-stratified and cell-type-specific investigations, is essential for identifying optimal therapeutic windows [161].

### 4.9. The PVAT–MAMs Axis in Atherosclerosis: A Dynamic and Multiscale Conceptual Framework

Although the PVAT–MAMs framework primarily emphasizes local interactions between perivascular adipose tissue and vascular cells, these processes are embedded within a broader immunometabolic network spanning multiple biological scales.

At the local level, immune cells within PVAT critically regulate adipose tissue phenotype and inflammatory signaling. Macrophages, which are abundant in dysfunctional PVAT, undergo dynamic polarization between pro-inflammatory and anti-inflammatory states, secreting cytokines, chemokines, and lipid mediators that may influence mitochondrial function, calcium signaling, and oxidative stress in neighboring vascular cells [4,162]. Other immune populations, including T cells, mast cells, and eosinophils, likely contribute to this regulatory milieu, although their specific roles in modulating MAMs remain to be explored.

Beyond local immunity, systemic physiological signals also shape the PVAT–MAMs axis. Neurohumoral factors (e.g., sympathetic nervous system activity) influence PVAT lipolysis, adipokine secretion, and inflammation [163,164]. Circulating hormones and metabolic regulators, including insulin, glucocorticoids, leptin, and adiponectin, modulate mitochondrial metabolism and ER stress responses in vascular cells [165]. Additionally, gut microbiota-derived metabolites such as short-chain fatty acids and trimethylamine-N-oxide have been implicated in vascular inflammation and mitochondrial dysfunction, suggesting microbiome-derived signals may indirectly affect ER–mitochondria communication [166].

Inter-organ metabolic crosstalk further shapes this axis. Organs that are central to metabolic regulation, which include the liver, pancreas, and gut, influence systemic lipid flux, inflammatory tone, and endocrine signaling [167,168]. For example, hepatic lipid metabolism and insulin resistance alter circulating lipid profiles that affect PVAT biology [169], while pancreatic endocrine dysfunction exacerbates vascular metabolic stress via dysregulated insulin and glucagon secretion [170,171]. Adipose tissue depots elsewhere in the body may also communicate with perivascular depots through endocrine signals, creating a network of inter-adipose crosstalk that influences local PVAT phenotype.

These interconnected pathways highlight that PVAT–MAMs interactions are embedded within a broader network linking metabolic organs, immune regulation, and vascular homeostasis. Recognizing this multiscale architecture may explain why metabolic disorders such as obesity and type 2 diabetes exert profound effects on vascular inflammation and atherosclerosis progression. It also underscores that therapeutic strategies targeting the PVAT–MAMs axis must account for these systemic modifiers to achieve durable, context-appropriate effects.

## 5. Pharmacological Modulation of the PVAT–MAMs Axis: A Conceptual and Exploratory Framework

The elucidation of the PVAT–MAMs axis provides a conceptual framework for organizing existing and emerging therapeutic strategies across biological scales. Rather than defining a validated therapeutic pathway, this framework highlights two interrelated pathogenic nodes: dysfunctional PVAT as an upstream signal source and dysregulated MAMs as a downstream intracellular integration hub. Conceptually, interventions that attenuate pathological signaling at the tissue level while stabilizing stress integration at the subcellular level may offer a rational approach to interrupt self-reinforcing inflammatory, lipotoxic, and oxidative processes in AS. Importantly, most strategies discussed below were not developed to specifically target PVAT or MAMs, and their effects on these compartments are largely indirect, pleiotropic, and predominantly supported by preclinical or associative evidence.

### 5.1. Upstream Intervention: Modulating PVAT Phenotype

#### 5.1.1. Foundational Lifestyle Modifications

Lifestyle interventions remain a foundational and well-supported approach for improving PVAT function. Regular aerobic exercise and low-fat, high-fiber diets have been shown to reduce PVAT inflammation, limit lipid overload, and partially restore adipokine secretion, thereby attenuating adverse paracrine effects on the vascular wall [27,172]. Mechanistic studies in animal models indicate that exercise activates the AMPK–Akt–eNOS axis within PVAT, promoting mitochondrial function and suppressing pro-inflammatory signaling [173]. These non-pharmacological strategies provide a baseline for modifying PVAT-derived paracrine signaling. However, the extent to which these interventions specifically modulate PVAT-derived signaling to vascular cells remains incompletely defined.

#### 5.1.2. Pharmacological Reprogramming

Glucagon-Like Peptide-1 (GLP-1) receptor agonists target PVAT by binding adipocyte GLP-1 receptors to improve its phenotype [159,174]. These drugs enhance the PI3K/Akt pathway to reduce lipolysis, suppress NF-κB–mediated inflammation, and increase adiponectin and UCP1 levels [175,176]. The net effect is a shift toward a “beige” anti-inflammatory PVAT phenotype with better thermogenic and lipid-clearing capacity. However, direct causal evidence linking PVAT-localized GLP-1R signaling to downstream MAMs remodeling in vascular cells remains limited.

PPARγ agonists modulate PVAT by enhancing mitochondrial function, promoting adipokine secretion, and repressing inflammatory gene expression [177,178]. Experimental studies suggest that improvements in vascular function can occur alongside PVAT microenvironment normalization, even in the absence of major changes in fat mass [48]. These findings underscore the functional relevance of PVAT modulation in vascular pathology, although they do not establish PVAT as a discrete or exclusive pharmacological target.

SGLT2 inhibitors have also been reported to attenuate oxidative stress and inflammation in PVAT while improving mitochondrial efficiency in experimental settings [179,180]. Clinically, reductions in epicardial adipose tissue volume have been observed following empagliflozin treatment [181,182], suggesting indirect modulation of perivascular fat depots.

Beyond their primary indications, several agents exert pleiotropic effects on PVAT. SIRT1 agonists such as resveratrol activate PGC-1α, improve mitochondrial redox balance, and reduce oxidative stress in adipose tissue [183,184]. These changes may indirectly influence stress signaling pathways converging on vascular cells, although direct links to MAMs regulation remain to be demonstrated. Antioxidants decrease PVAT oxidative stress and inflammation [185], and statin therapy has been shown to improve PVAT phenotype in patients [186,187].

### 5.2. Downstream Intervention: Targeting MAMs-Associated Stress Integration

#### 5.2.1. Calcium Signaling Modulation

Sig-1R agonists stabilize the IP_3_R–GRP75–VDAC1 complex, thereby supporting ER–mitochondrial Ca^2+^ homeostasis and limiting downstream stress signaling [188,189]. In contrast, GRP75 inhibitors actually disrupt ER–mitochondrial Ca^2+^ flux and have been shown to reduce VSMC calcification [190]. New, less toxic GRP75 inhibitors are being developed for precise MAMs modulation [191].

#### 5.2.2. Correction of Lipid Dysregulation

ACAT1 inhibitors suppress cholesterol esterification within macrophage MAMs, thereby limiting foam cell formation at an early intracellular stage [192]. Preclinical studies suggest synergistic plaque-stabilizing effects when combined with statins [193]. In parallel, experimental modulation of ORP5/8-mediated phospholipid exchange has been proposed to support endothelial membrane integrity and ER–mitochondrial lipid balance, although these strategies remain largely exploratory [129,194].

#### 5.2.3. Suppression of Inflammatory Signaling

Given the localization of NLRP3 inflammasome assembly at MAMs, inhibitors such as MCC950 have been used experimentally to attenuate inflammatory amplification [195]. Additionally, inhibition of the redox-sensitive protein TXNIP prevents its stress-induced translocation to MAMs and subsequent NLRP3 activation, thereby interrupting ROS–inflammation feedforward loops in experimental models [196,197].

#### 5.2.4. Structural Support of MAMs Integrity

Strengthening MAMs tethers is another strategy. For example, AAV-mediated MFN2 overexpression or MFN2-agonist compounds can increase ER–mitochondrial contacts [198,199,200]. Modulation of PERK activity at MAMs, particularly limiting its pathological overactivation while preserving adaptive signaling, has also been proposed as a future avenue for stabilizing MAMs function under chronic stress conditions.

### 5.3. Conceptual Rationale for Combined Multi-Target Approaches

Targeting a single node within the PVAT–MAMs axis may be insufficient to disrupt interconnected pathogenic feedback loops. From a conceptual perspective, combined strategies that attenuate PVAT-derived stress signals while limiting their intracellular amplification at MAMs may offer greater robustness. Such dual-level modulation aims to limit both the initiation and amplification of inflammatory, lipotoxic, and oxidative signals across biological scales. Importantly, these approaches remain hypothesis-driven and exploratory, and their efficacy, safety, and translational feasibility require rigorous validation.

### 5.4. Safety Considerations in Targeting the PVAT–MAMs Axis

While the PVAT–MAMs axis offers an attractive conceptual framework for therapeutic intervention, the safety and translational feasibility of targeting these pathways require careful consideration. Many of the molecular regulators discussed above, including Sig-1R, MFN2, PERK, and the NLRP3 inflammasome, participate in fundamental cellular processes such as calcium signaling, mitochondrial dynamics, lipid metabolism, and innate immune responses. Because these pathways are broadly expressed across multiple tissues and organ systems, systemic pharmacological modulation may carry the risk of unintended off-target effects.

For example, Sig-1R is highly expressed in the central nervous system and plays critical roles in neuronal survival and synaptic signaling [201]. Consequently, systemic modulation of Sig-1R activity may potentially influence neurological function. Similarly, MFN2 serves as a key regulator of mitochondrial fusion and metabolic homeostasis, and excessive or prolonged manipulation of MFN2 signaling could disrupt mitochondrial dynamics in metabolically active tissues such as skeletal muscle, liver, or cardiac tissue [202]. Inhibition of inflammatory mediators such as the NLRP3 inflammasome may also compromise host defense mechanisms if immune responses are excessively suppressed [203]. These concerns are further compounded by pharmacologically manipulating pathways that are deeply embedded in core cellular physiology. Exacerbating these risks is the context-dependent dual role of MAMs, meaning that interventions aimed at inhibiting pathological functions may inadvertently block adaptive responses, particularly when applied at the wrong disease stage. Furthermore, achieving spatial and temporal precision remains a major obstacle. Therefore, rigorous verification of their biodistribution, safety, and durability is required before clinical applications can be considered.

## 6. Challenges and Future Directions

### 6.1. Establishing Causality

Current evidence supporting the PVAT–MAMs axis is predominantly associative and largely derived from in vitro systems or rodent models, thereby limiting definitive conclusions regarding directionality, necessity, or causal sufficiency in AS. In humans, evidence remains largely observational, including histological characterization of PVAT and imaging-derived biomarkers, without direct demonstration of PVAT–MAM functional coupling. Consequently, a fundamental question remains unresolved: Is PVAT dysfunction a primary driver of vascular pathology via MAMs remodeling, a secondary consequence of plaque-derived signals, or an epiphenomenon unrelated to the core disease mechanism? Determining whether this axis represents a mechanistic driver rather than an epiphenomenon therefore requires rigorous in vivo validation.

A definitive approach would involve bidirectional Cre–Lox-based manipulation: adipocyte-restricted deletion or overexpression of candidate PVAT-derived mediators (e.g., pro-inflammatory cytokines, FFAs) followed by vascular assessment of MAM architecture and function, and conversely, vascular cell–specific targeting of canonical MAM tethering proteins such as MFN2 or PACS2 in otherwise healthy or metabolically stressed animals would determine whether disruption of organelle contact integrity modifies the vascular consequences of PVAT dysfunction. Such strategies enable formal testing of necessity and sufficiency, particularly when combined with inducible systems and lineage tracing to resolve temporal hierarchy [204]. This bidirectional approach is essential to distinguish whether MAMs are a necessary conduit for PVAT signals or merely a downstream bystander.

Mechanistic specificity further requires direct quantification of ER–mitochondria contact remodeling rather than inference from global cellular stress markers. Proximity ligation assays targeting tethering complexes, super-resolution or electron microscopy-based structural analyses, and organelle-targeted calcium biosensors assessing interorganelle flux provide spatially and functionally resolved endpoints [205,206]. Importantly, these measurements must be performed in a cell-type-specific manner (e.g., VSMCs vs. ECs) within the intact vessel wall to capture the heterogeneity of cellular responses. Complementary lipid or metabolite tracing approaches may clarify whether PVAT-derived substrates accumulate at or modulate MAM-associated signaling nodes.

Translation beyond rodent systems is essential. In human tissues, ex vivo PVAT–vessel co-culture models and biopsy-based structural assays may provide initial validation [207]. Emerging organ-on-a-chip technologies incorporating human PVAT and vascular cells under flow conditions could offer a controlled platform to dissect human-relevant mechanisms [208].Advanced metabolic imaging modalities combined with coronary metrics such as Fat Attenuation Index (FAI) could offer indirect but scalable assessments of PVAT–vascular coupling, contingent upon careful validation against histological endpoints [204,209]. Collectively, these approaches may shift the PVAT–MAMs axis from conceptual association toward experimentally substantiated causality. However, the development of circulating or imaging-based surrogate markers specifically reflecting vascular MAMs dysfunction remains a critical unmet need for clinical translation. Until such evidence is obtained, the framework should be regarded as a testable hypothesis that identifies key nodes for mechanistic interrogation rather than an established pathogenic pathway.

Time-resolved experimental designs tracking the natural history of atherosclerosis from early metabolic stress to advanced plaque formation will be critical to determine whether PVAT inflammation precedes MAM disruption or represents a secondary amplification mechanism. Moreover, these processes likely exhibit cell-type-specific heterogeneity, with endothelial cells and vascular smooth muscle cells potentially responding to PVAT-derived signals on different timescales. Capturing this dynamic complexity requires spatiotemporally resolved methodologies that move beyond static snapshots. Future studies should integrate: Longitudinal animal models with sampling at defined disease stages (e.g., early vs. advanced plaque) [210]. Spatial transcriptomics and proteomics to map molecular changes across tissue compartments (from PVAT to intima) and cell types [211,212]. High-resolution imaging of ER–mitochondria contact sites (e.g., electron microscopy, PLA) with cell-type-specific resolution [91]. Stage-stratified human tissue analyses correlating PVAT inflammatory status with ultrastructural evidence of MAM remodeling [213,214]. Such a spatiotemporal framework is essential to distinguish whether PVAT–MAMs interactions shift from adaptive metabolic communication in early disease to maladaptive inflammatory signaling in advanced AS, a distinction that carries profound implications for stage-specific therapeutic targeting.

### 6.2. Biological and Clinical Heterogeneity

The PVAT–MAMs axis should not be viewed as a spatially uniform or biologically invariant mechanism. Rather, its relevance and manifestation are likely shaped by anatomical location, species background, biological age, and patient-specific metabolic context. A critical dimension of this heterogeneity is the anatomical location of the vascular bed. PVAT depots exhibit marked regional diversity in thermogenic capacity, immune composition, and lipid handling [215]. Thoracic PVAT, which exhibits brown-like characteristics in rodents, may support mitochondrial homeostasis and MAM stability [4,216]. In contrast, abdominal or coronary PVAT, characterized by greater white adipocyte predominance and macrophage accumulation, may favor lipotoxic stress, mitochondrial cholesterol loading, and maladaptive MAM remodeling [9,217]. For example, coronary PVAT is metabolically active and closely associated with inflammatory signaling that directly influences coronary atherosclerosis, whereas PVAT surrounding the thoracic aorta often displays distinct structural and thermogenic characteristics [218]. Similarly, abdominal aortic PVAT has been reported to exhibit greater lipid accumulation and inflammatory activation in metabolic disease states [219]. These regional differences suggest that the strength, directionality, and pathological consequences of PVAT-derived signaling may vary depending on the vascular bed, local metabolic environment, and disease context. Vascular cells themselves also exhibit location-specific phenotypes that may modulate MAMs responses. ECs and VSMCs derived from different vascular beds display distinct metabolic profiles, inflammatory responses, and mitochondrial dynamics [220], which could influence ER–mitochondria communication and either amplify or attenuate the impact of PVAT-derived signals depending on the vascular territory.

Temporal and patient-level factors further complicate this framework [221]. Aging independently remodels PVAT phenotype and mitochondrial tethering dynamics [222], impairing ER–mitochondria coupling under metabolic stress. Sex differences, genetic predisposition, insulin resistance, dyslipidemia, and comorbid conditions may modify both PVAT inflammatory tone and organelle vulnerability [111,223]. Furthermore, genetic and epigenetic mechanisms may contribute to interindividual variability in the PVAT–MAMs axis. Genetic variants affecting mitochondrial function, inflammatory signaling, or lipid metabolism could influence the susceptibility of vascular cells and PVAT to metabolic stress, potentially modulating mitochondrial dynamics, ER stress responses, and inflammatory pathways that converge at MAMs [224,225]. Epigenetic regulation provides an additional layer of control. DNA methylation, histone modifications, and non-coding RNAs have been shown to influence adipose tissue inflammation, mitochondrial metabolism, and vascular cell phenotype in cardiometabolic diseases [226,227]. These epigenetic marks may dynamically regulate gene expression within PVAT and vascular cells in response to environmental cues such as diet, metabolic status, or systemic inflammation, thereby shaping the functional state of the PVAT–MAMs axis over time. Understanding these genetic and epigenetic determinants will be essential for moving toward personalized approaches to risk stratification and therapeutic intervention, as they may define patient subgroups with differential susceptibility to PVAT–MAMs-mediated vascular pathology.

Future studies region-specific sampling strategies, spatially resolved omics approaches, and longitudinal cohorts with stratified clinical analyses will be necessary to determine whether the PVAT–MAMs axis defines specific endophenotypes of atherosclerosis rather than representing a universally dominant mechanism [228,229]. Comparing PVAT–vascular interactions across anatomical regions will be essential for determining the generalizability of this framework and for identifying vascular territories in which PVAT–MAMs signaling may play a particularly prominent role in atherosclerosis progression. Importantly, the impact of the PVAT–MAM axis is likely stage-dependent, with early adaptive ER–mitochondria responses potentially evolving into maladaptive Ca^2+^ dysregulation and inflammatory amplification in advanced disease, underscoring the need for temporally resolved and stage-stratified investigation. Accordingly, the axis may exert greater influence during plaque progression and vulnerability rather than initial lipid deposition, although this hierarchy remains to be formally tested.

### 6.3. Translational Barriers and Opportunities

Despite its conceptual appeal, translation of the PVAT–MAMs axis faces substantial challenges. First, Importantly, direct evidence demonstrating the PVAT–MAMs interaction in human vascular tissues remains extremely limited. First and foremost, a fundamental translational gap exists between rodent models and human biology. The majority of mechanistic insights underpinning the PVAT–MAMs framework derive from murine studies. However, human PVAT exhibits critical differences, including reduced thermogenic plasticity, distinct immune cell composition (e.g., lower eosinophil and higher macrophage content in certain depots), and a more limited capacity for “beiging” compared to rodent PVAT [221,230]. Consequently, the protective metabolic buffering observed in murine thoracic PVAT models may be substantially attenuated in humans. Furthermore, human vascular cells may display different stress responses and MAMs dynamics. These interspecies disparities mean that findings in mice cannot be directly extrapolated to human pathophysiology. Bridging this gap requires a dedicated pipeline of human-relevant experimental approaches: Ex vivo human tissue platforms, such as co-cultures of human PVAT explants (obtained from cardiac surgery or tissue banks) with primary human vascular cells or arterial segments, can directly test whether human perivascular signals recapitulate the MAMs-directed effects observed in animal models [231]. Human biopsy and autopsy studies, coupled with high-resolution imaging (e.g., electron microscopy of vascular specimens from patients across the spectrum of atherosclerosis), are essential to establish whether PVAT inflammatory status correlates with ultrastructural evidence of MAM remodeling in the human vessel wall [232]. Induced pluripotent stem cell-derived vascular cells and adipocytes from genetically diverse patient cohorts offer a controlled, human-specific platform to dissect the axis and explore inter-individual variability [233]. The development of circulating or EV-based biomarkers that reflect vascular MAMs stress (e.g., MAM-resident proteins, specific lipid species) is critical, as these could be measured in patient plasma and correlated with PVAT imaging metrics, enabling non-invasive assessment of the axis in living humans [154].

Second, the axis operates within a broader inter-organelle and intercellular signaling network whose hierarchy and feedback topology remain incompletely defined, including contributions from cytokines such as IL-1β and IL-18, interferon signaling, diverse lipid mediators, and neurohormonal influences [234]. Systems-level approaches integrating single-cell and spatial omics, quantitative imaging, and functional perturbation platforms will be required to distinguish primary drivers from compensatory or emergent responses [235,236,237]. Quantitative network modeling and systems biology approaches will be necessary to assess signal dominance, feedback strength, and emergent properties. Moreover, systemic modifiers, including hemodynamic forces such as blood pressure, neurohormonal signaling pathways, and gut microbiota-derived metabolites, may converge on both PVAT phenotype and vascular MAM dynamics, further emphasizing that the proposed axis operates within a broader integrative physiological network rather than as an isolated pathway.

Third, therapeutic targeting necessitates spatial precision. Modulation of PVAT inflammation or MAM integrity must achieve compartment-specific effects while minimizing systemic toxicity. Strategies such as adipose-tropic delivery systems or mitochondria-directed conjugates remain exploratory and require rigorous validation of biodistribution, safety, and durability before clinical application can be considered [238,239]. Moreover, biological compensation and pathway redundancy may attenuate single-node interventions, complicating dose–response prediction and long-term efficacy. Given the central role of calcium signaling, lipid metabolism, and redox regulation in normal cellular physiology, excessive or non-specific modulation of MAM-associated pathways may carry unintended systemic consequences.

Fourth, effective clinical translation depends on reliable biomarkers capable of capturing MAMs dysfunction in vivo. A key barrier to the clinical translation of the PVAT–MAMs axis is the lack of validated biomarkers that directly reflect MAMs dysfunction in vivo. Although direct visualization of ER–mitochondria contact sites is currently limited to ultrastructural or experimental approaches, several indirect molecular and imaging indicators may serve as potential surrogates. At the molecular level, circulating markers associated with mitochondrial stress and ER dysfunction, which include mitochondrial DNA, fibroblast growth factor-21, growth differentiation factor-15, and ER stress mediators such as C/EBP homologous protein and 78 kDa glucose-regulated protein, have been proposed as indicators of mitochondrial and ER homeostasis [240,241,242]. Tissue-level alterations in the expression of MAM-associated proteins (e.g., MFN2, PACS2, and Sig-1R) in vascular specimens may also reflect remodeling of ER–mitochondria contact sites. At the imaging level, emerging metrics such as the perivascular FAI derived from coronary computed tomography angiography indirectly capture PVAT inflammatory status and vascular metabolic stress, and may therefore serve as a surrogate for the upstream component of the PVAT–MAMs axis [243]. Integration of molecular biomarkers with advanced imaging approaches could provide a multimodal strategy to approximate PVAT–MAMs dysfunction in clinical settings. However, systematic validation in well-characterized longitudinal cohorts is required to establish their specificity, sensitivity, and predictive value for cardiovascular outcomes.

Finally, effective clinical translation depends on reliable patient stratification. Imaging-derived metrics such as coronary FAI provide non-invasive assessment of PVAT inflammatory status and have been associated with plaque activity and cardiovascular risk [244,245]. However, standardization, demographic variability, and cross-cohort validation remain critical prerequisites [246,247]. The dynamic behavior of these biomarkers under therapeutic intervention also remains insufficiently characterized. Integration of imaging markers with circulating indicators potentially reflecting mitochondrial or MAM dysfunction may offer a multimodal stratification framework, although such approaches remain investigational [248].

## 7. Conclusions

In this review, we propose the PVAT–MAMs axis as an integrative, cross-scale conceptual framework linking perivascular metabolic dysfunction with subcellular stress integration in vascular cells. By synthesizing accumulating experimental and associative evidence, this framework highlights a potential route through which metabolic disturbances in the perivascular microenvironment may influence intracellular vulnerability during atherosclerotic progression.

Nevertheless, the PVAT–MAMs axis should be viewed as one component of a broader pathogenic landscape rather than a unifying explanation for all forms of AS. Its relevance is likely to vary across vascular beds, disease stages, and metabolic contexts. Well-established pathogenic drivers, including luminal lipid infiltration, hemodynamic forces, and systemic immune activation, can operate independently of PVAT and remain central to disease initiation and progression. Accordingly, the PVAT–MAMs axis is best interpreted as a complementary perivascular perspective, rather than a replacement for classical paradigms.

Within these constraints, the proposed framework offers a conceptual basis for understanding disease heterogeneity. Variability in PVAT dysfunction may contribute to differential intracellular stress responses, thereby influencing plaque evolution and vulnerability in a context-dependent manner. In parallel, this perspective provides a mechanistic lens through which metabolic disorders such as obesity and type 2 diabetes may exacerbate atherosclerotic progression via persistent extravascular stress signaling.

We acknowledge that the PVAT–MAMs axis remains a hypothesis-driven model that requires rigorous causal validation. Future studies employing cell-type-specific genetic manipulation, advanced in vivo imaging, and human-relevant experimental systems will be essential to clarify the directionality, temporal sequence, and vascular-bed specificity of PVAT–MAMs communication.

Despite these limitations, this framework has important conceptual implications. It highlights PVAT and MAMs as interconnected, but not exclusive, pathogenic nodes and suggests that coordinated consideration of tissue-level metabolic stress and subcellular stress integration may inform future mechanistic studies. More broadly, by bridging systemic metabolism with intracellular signaling organization, the PVAT–MAMs axis provides a structured platform for generating testable hypotheses and guiding future research in AS. As our understanding of inter-organelle and inter-tissue communication evolves, the PVAT-MAMs axis should be interpreted not as an isolated linear pathway, but as a dynamic convergence node within a complex adaptive system, whose causal architecture remains to be empirically defined.

## Figures and Tables

**Table 1:** The Phenotypic and Functional Switch of PVAT from a Protective to a Pathogenic State under Metabolic Stress.

Functional Aspect	Physiological State of PVAT	Pathological State of PVAT	References
Phenotypic State	Beiging Phenotype: (a) Adipocytes are rich in mitochondria. (b) High expression of UCP1. (c) Strong thermogenic and metabolic activity.	Whitening Phenotype: (a) Adipocyte hypertrophy with LD fusion. (b) Mitochondrial dysfunction and reduced UCP1. (c) Exhibits a state of metabolic disorder.	[6,16,17,24,25]
Core Metabolic Regulators	Dominant PPARγ/PGC-1α Signaling: (a) Highly activated, driving mitochondrial biogenesis. (b) Promotes FFA β-oxidation. (c) Positively regulates UCP1 to maintain the thermogenic phenotype.	Impaired Protective Signaling: (a) In Obesity: Induces abnormal HIF-1α accumulation. (b) In T2D: The AGEs-RAGE pathway inhibits PPARγ function. (c) Collective failure to maintain the beiging phenotype.	[18,27,28,29,30]
Paracrine Factors & Roles	Secretion of Protective Factors: (a) Vasodilation: Releases ADRFs, activating the cGMP/PKG pathway. (b) Anti-inflammatory & Metabolic Regulation: Secretes APN and FGF21, inhibiting inflammation and foam cell formation.	Secretion of Pathogenic Factors: (a) Pro-inflammatory Amplification: Secretes IL-6, TNF-α, and AGEs, activating AGEs-RAGE/TLR4 pathways. (b) Vascular Dysfunction: FFA overflow impairs endothelium-dependent vasodilation. (c) Hormonal Imbalance: Decreased APN and increased leptin disrupt the pro-/anti-inflammatory balance.	[19,20,21,24,25,31]
Effects on Vascular Cells	Holistic Vascular Protection: (a) On Macrophages: Inhibits ox-LDL uptake and foam cell formation. (b) On VSMCs: Maintains the contractile phenotype, promoting vessel stability. (c) On ECs: Maintains endothelial integrity and NO bioavailability, promoting anti-inflammatory and anti-thrombotic functions.	Driving Atherogenic Processes: (a) On Macrophages: Promotes infiltration and foam cell formation. (b) On VSMCs: Induces a switch to the synthetic phenotype, upregulating MMP-2/9 and weakening the plaque fibrous cap. (c) On ECs: Induces endothelial dysfunction, increasing permeability, adhesion molecule expression, and pro-thrombotic state.	[29,30,31,32,33,34,35,36]
Metabolic Buffering Capacity	Efficient and Safe Lipid Processor: (a) Efficient Clearance: Metabolizes circulating FFAs via high-capacity β-oxidation. (b) Dynamic Balance: Maintains lipolysis/re-esterification balance via ATGL. (c) Protective Barrier: Prevents lipotoxic injury to ECs and VSMCs.	Lipid Dysregulation and Toxicity: (a) Lipid Overflow: Obesity causes excessive FFA release; T2D impairs oxidation; LD instability. (b) Direct Injury: Overflowing FFAs cause direct lipotoxic damage to ECs and VSMCs.	[22,23,37]

Lipid Droplet(LD), Advanced glycation end products and their receptor (AGEs-RAGE),Cyclic Guanosine Monophosphate/Protein Kinase G signaling pathway (cGMP/PKG), Toll-like receptor 4 (TLR4), Adipose Triglyceride Lipase (ATGL), Protein Kinase C (PKC) Hypoxia-Inducible Factor-1α (HIF-1α), Oxidized Low-Density Lipoprotein (ox-LDL), Matrix Metalloproteinase-2/9(MMP-2/9), Vascular Smooth Muscle Cells (VSMCs),Endothelial Cells(ECs).

## Data Availability

No new data were created or analyzed in this study. Data sharing is not applicable to this article.

## Nonstandard Abbreviations and Acronyms

AS	Atherosclerosis
PVAT	Perivascular Adipose Tissue
MAMs	Mitochondria-Associated Endoplasmic Reticulum Membranes
T2D	Type 2 Diabetes
FFAs	Free Fatty Acids
ROS	Reactive Oxygen Species
WAT	White Adipose Tissue
BAT	Brown Adipose Tissue
BeAT	Beige Adipose Tissue
UCP1	Uncoupling Protein 1
PPARγ	Peroxisome Proliferator-Activated Receptor γ
PGC-1α	PPARγ Coactivator-1α
ADRFs	Adipose-Derived Relaxing Factors
APN	Adiponectin
NO	Nitric Oxide
FGF21	Fibroblast Growth Factor 21
NRG4	Neuregulin 4
AGEs	Advanced Glycation End Products
RAGE	Receptor for Advanced Glycation End Products
IL-6	Interleukin-6
TNF-α	Tumor Necrosis Factor-α
HIF-1α	Hypoxia-Inducible Factor-1α
TLR4	Toll-Like Receptor 4
LD	Lipid Droplet
cGMP/PKG	Cyclic Guanosine Monophosphate/Protein Kinase G Signaling Pathway
ATGL	Adipose Triglyceride Lipase
PKC	Protein Kinase C
ox-LDL	Oxidized Low-Density Lipoprotein
ECs	Endothelial Cells
IL-1β	Interleukin-1β
eNOS	Endothelial Nitric Oxide Synthase
NOX	NADPH Oxidase
VSMCs	Vascular Smooth Muscle Cells
MMP-2/9	Matrix Metalloproteinase-2/9
SIRT1	Silent Information Regulator 2 Homolog 1
LOX-1	Lectin-Like Oxidized Low-Density Lipoprotein Receptor-1
Piezo1	Piezo Type Mechanosensitive Ion Channel Component 1
YAP/TAZ	Yes-Associated Protein/Transcriptional Coactivator With PDZ-Binding Motif
NF-κB	Nuclear Factor Kappa B
VDAC	Voltage-Dependent Anion Channel
IP_3_R2	Inositol 1,4,5-Trisphosphate Receptor
MDA	Malondialdehyde
IP_3_R	Inositol 1,4,5-Trisphosphate Receptor
GRP75	Glucose-Regulated Protein 75
ACSL4	Acyl-CoA Synthetase 4
PERK	Protein Kinase R-Like Endoplasmic Reticulum Kinase
Sig-1R	Sigma-1 Receptor
ORP5/8	Oxysterol-Binding Protein-Related Protein 5 and 8
PS	Phosphatidylserine
PC	Phosphatidylcholine
ACAT1	Acyl-CoA:Cholesterol Acyltransferase 1
FC	Free Cholesterol
CE	Cholesteryl Ester
mtROS	Mitochondrial Reactive Oxygen Species
GRAMD1C	GRAM Domain Containing 1C
ERS	Endoplasmic Reticulum Stress
mPTP	Mitochondrial Permeability Transition Pore
MFN2	Mitofusin 2
VAPB	Vesicle-Associated Membrane Protein-Associated Protein B
Drp1	Dynamin-Related Protein 1
PINK1	PTEN-Induced Kinase 1
Parkin	Parkin RBR E3 Ubiquitin Protein Ligase
FUNDC1	FUN14 Domain Containing 1
ACADM	Acyl-CoA Dehydrogenase Medium Chain
PACS2	Phosphofurin Acidic Cluster Sorting Protein 2
STAT3	Signal Transducer and Activator of Transcription 3
caspase-1	Cysteine–Aspartic Acid Protease-1
CD36	Cluster of Differentiation 36
NLRP3	NOD-Like Receptor Protein 3
PTPIP51	Protein Tyrosine Phosphatase Interacting Protein 51
ASC	Apoptosis-Associated Speck-Like Protein Containing a CARD
IRE1α	Inositol-Requiring Enzyme 1α
PI4P	Phosphatidylinositol 4-Phosphate
VE-cadherin	Vascular Endothelial Cadherin
CaN	Calcineurin
NFATc3	Nuclear Factor of Activated T Cells, Cytoplasmic 3
4HNE	4-Hydroxynonenal
AMPK	AMP-Activated Protein Kinase
GPX4	Glutathione Peroxidase 4
GSH	Glutathione
TXNIP	Thioredoxin-Interacting Protein
GLP-1	Glucagon-Like Peptide-1
PI3K/Akt	Phosphoinositide 3-Kinase/Protein Kinase B
HSL	Hormone-Sensitive Lipase
SGLT2	Sodium-Glucose Cotransporter 2
AAV	Adeno-Associated Virus
PET–CT	Positron Emission Tomography–Computed Tomography
MRI	Magnetic Resonance Imaging
sRAGE	Soluble Receptor for Advanced Glycation End Products
MKT-077	A Small-Molecule Inhibitor of GRP75
MCC950	A Small-Molecule Inhibitor of NLRP3
KO	Knockout
scRNA-seq	Single-Cell RNA Sequencing
FAI	Fat Attenuation Index
